# A model for boundary-driven tissue morphogenesis

**Published:** 2025-03-05

**Authors:** Daniel S. Alber, Shiheng Zhao, Alexandre O. Jacinto, Eric F. Wieschaus, Stanislav Y. Shvartsman, Pierre A. Haas

**Affiliations:** 1 Department of Chemical and Biological Engineering, Princeton University, Princeton, NJ 08540; 2 The Lewis-Sigler Institute for Integrative Genomics, Princeton University, Princeton, NJ 08540; 3 Max Planck Institute for the Physics of Complex Systems, Nöthnitzer Strae 38, 01187 Dresden, Germany; 4 Max Planck Institute of Molecular Cell Biology and Genetics, Pfotenhauerstrae 108, 01307 Dresden, Germany; 5 Center for Systems Biology Dresden, Pfotenhauerstrae 108, 01307 Dresden, Germany; 6 Center for Computational Biology, Flatiron Institute, Simons Foundation, New York, NY 10010; 7 Department of Molecular Biology, Princeton University, Princeton, NJ 08540

## Abstract

Tissue deformations during morphogenesis can be active, driven by internal processes, or passive, resulting from stresses applied at their boundaries. Here, we introduce the *Drosophila* hindgut primordium as a model for studying boundary-driven tissue morphogenesis. We characterize its deformations and show that its complex shape changes can be a passive consequence of the deformations of the active regions of the embryo that surround it. First, we find an intermediate characteristic triangular shape in the 3D deformations of the hindgut. We construct a minimal model of the hindgut primordium as an elastic ring deformed by active midgut invagination and germ band extension on an ellipsoidal surface, which robustly captures the symmetry-breaking into this triangular shape. We then quantify the 3D kinematics of the tissue by a set of contours and discover that the hindgut deforms in two stages: an initial translation on the curved embryo surface followed by a rapid breaking of shape symmetry. We extend our model to show that the contour kinematics in both stages are consistent with our passive picture. Our results suggest that the role of in-plane deformations during hindgut morphogenesis is to translate the tissue to a region with anisotropic embryonic curvature and show that uniform boundary conditions are sufficient to generate the observed nonuniform shape change. Our work thus provides a possible explanation for the various characteristic shapes of blastopore-equivalents in different organisms and a framework for the mechanical emergence of global morphologies in complex developmental systems.

## I. INTRODUCTION

Morphogenesis can proceed through active mechanisms, which generate tissue deformations by changing cell behaviors within their bounds, or passive mechanisms, which generate deformations via external conditions imposed at their boundaries by neighboring tissues [1]. The interplay between active and passive tissues is particularly important during gastrulation, when an embryo has multiple genetically patterned active tissues in addition to passive regions that all deform significantly and almost simultaneously [2].

**FIG. 1.**
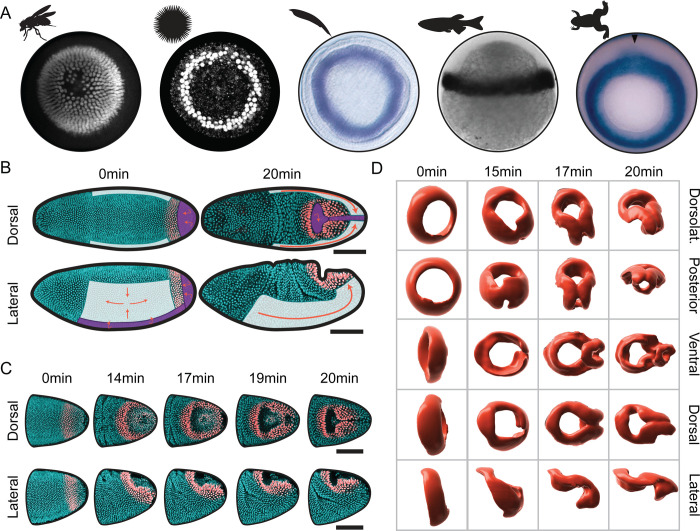
The hindgut primordium is bounded by active tissues and rapidly deforms in 15 minutes. (A) *brachyury* ortholog expression at the gastrula stage across the animal kingdom, in (left to right) the fruit fly *Drosophila melanogaster* [7], the sea urchin *Lytechnius variegatus* [27], the lancelet *Branchiostoma floridae* [28], the vertebrate ray-finned zebrafish *Danio rerio* [23], and the amphibian frog *Xenopus laevis* [26]. (B) Dorsal and lateral views of the blastoderm at the onset of gastrulation and 21 minutes later. The cyan signal is a nuclear reporter and the red signal is a nuclear reporter specific to the hindgut (Materials and Methods). The germ band, which undergoes in-plane convergent extension, is shaded in white. The ventral furrow and the posterior midgut undergo out-of-plane invagination and are shaded in purple. (C) Dorsal (top) and lateral (bottom) views of the deforming hindgut primordium at five timepoints, showing invagination of the posterior midgut as the hindgut deforms into its characteristic triangular shape. (D) Different views of surface reconstructions of the hindgut primordium from fixed data at timepoints approximated by morphology. Scale bars: 100 μm.

Perhaps no developmental system is as well understood as the *Drosophila melanogaster* embryo at the onset of gastrulation, composed of a monolayer of maternally patterned cells between an internal yolk and a vitelline membrane encapsulated by an ellipsoidal rigid chorion. At this stage, several canonical examples of active tissues that are genetically patterned to induce changes in cell shape or activity are undergoing morphogenesis: the posterior midgut (PMG), the ventral furrow (VF), and the germ band (GB) (3, 4, Fig. 1B). At the posterior pole, the PMG expresses the transcription factors Huckebein and Tailless [5–7] that signal through the GPCR ligand Fog to activate myosin and induce apical constriction and invagination of the posterior [8–10]. Similarly, a stripe of cells in the VF undergoes apical constriction and invaginates to form the mesoderm [11–13]. In addition to these out-of-plane deformations, the GB undergoes directed cell-cell rearrangements to converge and extend in-plane, pushing posterior tissue around the posterior pole onto the dorsal side of the embryo [14–18].

While the deformations of these active tissues are striking, they are separated at their boundaries by a domain of cells that deforms no less dramatically, although it lacks obvious expression of genes regulating active deformation [7]. This circular domain will ultimately give rise to the hindgut and consists of approximately 450 cells expressing Brachyenteron (*Drosophila* Brachyury). Brachyenteron defines a highly conserved signaling module specifying the posterior fates and gut formation in many organisms [19–22]. Homologs include T in mouse, No-tail in zebrafish, and XBra in *Xenopus*, and are typically present at the lip of the blastopore-equivalent posterior internalization (23–28, Fig. 1A). In *Drosophila*, the domain is ring-shaped and located anterior to the PMG but posterior to the VF and GB (7, Fig. 1B). Although Brachyenteron expression is ultimately required for cell-fate-specific differentiation of the hindgut, its elimination has no direct effect on the morphogenetic movements that occur at gastrulation [29]. This raises the possibility that early morphogenesis in the hindgut is imposed by forces generated in the surrounding regions.

Embryos provide numerous examples of active deformations in one region exerting forces on neighboring primordia, possibly contributing to their subsequent morphogenesis. Examples of such “boundary-driven” deformations include differential tissue growth driving brain gyrification [30] and vertebrate gut looping [31–33], friction forces driving the first folding event of the zebrafish brain [34, 35] and myotome formation [36], and active contractility at the tissue boundary driving amniote embryogenesis [37, 38]. A large body of work has characterized the diverse cellular processes that arise in response to external forces [39], adjacent domains [40, 41], and geometric constraints [42–46], including at the level of individual contributions within a tissue exhibiting both active and passive cellular behaviors [41, 47, 48]. However, explanations for global morphological changes of entire passive tissues in the necessary context of their active neighbors and geometric constraints have remained elusive. The *Drosophila* hindgut primordium offers an ideal system to develop a framework for understanding the deformations of such a passive tissue.

In the following experiments, we derive a minimal physical model to investigate whether contributions from adjacent actively-deforming tissues and embryonic geometry are sufficient to explain the morphogenesis of the hindgut primordium. We couple our model with 3D imaging of live embryos to quantify the deformations of the hindgut primordium rigorously. We find that as the PMG, VF, and GB impose forces at the boundary of the hindgut primordium, the primordium itself deforms in a combination of in-plane and out-of-plane deformations, breaking the symmetry of its circular shape into a characteristic, intermediate triangular “keyhole” shape (Fig. 1C,D). By tracking cells, we reveal a two-stage process and show that the kinematics of both stages are consistent with the passive deformations expected from forces applied at its boundary by the extension of the germ band and the invagination of the midgut that surround it.

## II. RESULTS

### Description of hindgut deformation at discrete timepoints

To visualize the deformations of the hindgut primordium, we used an endogenous fluorescent Brachyenteron protein reporter built on the LlamaTag system [7, 49] to identify the hindgut primordium combined with a standard fluorescently tagged histone nuclear reporter to visualize the entire embryo. Briefly, the LlamaTag system leverages maternally-deposited eGFP, which is imported to the nucleus upon the presence of a nanobody fused to the endogenous protein of interest (in our case, Brachyenteron). The deformations of the hindgut primordium were initially visualized using confocal microscopy (Materials and Methods).

At the onset of gastrulation, the initially circular tissue deforms significantly in a few minutes with no divisions nor cell death, and limited, if any, cell rearrangements (Fig. 1C). The ring initially rotates and translates along the surface of the embryo due to germ band extension (Fig. 1C, lateral views at 0–14 min) and partially internalizes due to contact with the apically constricting and invaginating posterior midgut (Fig. 1C, dorsal views at 0–14 min). After this initial phase, the ring rapidly deforms into a characteristic triangular “keyhole” shape (Fig. 1C, dorsal views at 17–20 min).

To create a more detailed description of these intermediate shapes, wildtype embryos were fixed, optically cleared, and stained for Brachyenteron and cell membrane markers Armadillo and Discs large. To visualize the deforming hindgut in 3D at a high isotropic spatial resolution, embryos were imaged using light sheet microscopy (Materials and Methods) and staged based on their morphology. Surface reconstructions from the Brachyenteron immunofluorescence signal (Fig. 1D) reveal complex intermediate geometries in which the internalized “keyhole” and the triangular shape of the tissue remaining on the surface are more apparent.

### Model of the symmetry-breaking of the hindgut

**FIG. 2.**
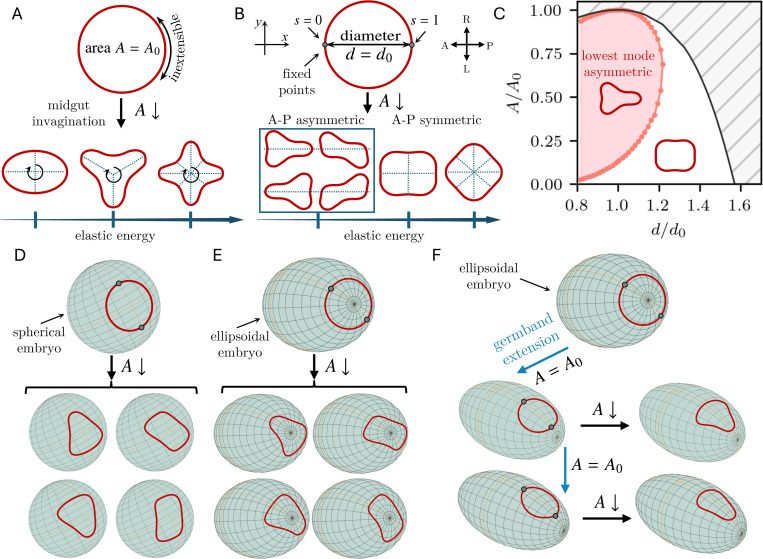
A minimal physical model reproduces the triangular keyhole shape of the primordial hindgut. (A) The primordial hindgut is modeled as a planar, inextensible elastic ring enclosing an initial area $A=A_{0}$. Invagination of the midgut reduces the enclosed area to A. The observed shape is the shape of lowest energy and symmetric [50, 51]. Additional modes with higher energy also exist and have higher numbers of lobes [50, 51]. (B) The position of the germ band additionally sets the anteroposterior (AP) diameter d of the ring, i.e., the distance between two diametrically opposite points at arclength positions s=0, s=1. For $d=d_{0}$, the four shapes of equal lowest energy are AP asymmetric, i.e., asymmetric about the y-axis, and include triangular shapes similar to the shape of the primordial hindgut. Additional symmetric and asymmetric shapes are possible as well, but are of higher energies (Materials and Methods). (C) Phase diagram of the bifurcation from panel (B) in (d, A) space: The AP asymmetric keyhole shape remains the lowest-energy mode in the shaded region of parameter space as A (midgut invagination) and d (germ band extension) vary. The hatched region is geometrically inaccessible to inextensible deformations. (D) An inextensible elastic ring constrained to lie on a sphere breaks symmetry into one of four shapes with equal energies, analogous to the planar shapes in panel (B), as the area enclosed by the ring is reduced (midgut invagination) while a diameter is fixed (germ band extension). (E) An elastic ring at the posterior pole of an ellipsoid embryo breaks symmetry similarly to the spherical case in panel (D). (F) Symmetry-breaking of an elastic ring at the posterior pole of an ellipsoid after translation to the dorsal side (germ band extension) and reduction of the area enclosed by the ring (midgut invagination): Among the shapes in panels (D), (E), the gradient in curvature consistently selects the triangular shape with the orientation observed in the *Drosophila* hindgut primordium.

We hypothesized that the shape changes of the hindgut primordium are the passive mechanical consequences of the deformations of the surrounding tissues. We therefore started by deriving a minimal theoretical model of hindgut morphogenesis. In this model, the hindgut primordium is skeletonized to a planar inextensible elastic ring 𝒞 enclosing an area occupied by the posterior midgut. The ring is initially circular, of area $A=A_{0}$ (Fig. 2A). As the midgut invaginates by apical constriction, the effective apical surface area of the tissue decreases, which reduces A and deforms the ring. This deformation minimizes the bending energy of the ring,

(1) $$𝓔=\frac{1}{2}\oint_{𝒞}\kappa {\left(s\right)}^{2}\text{d}s,$$

where s is arclength and κ(s) is curvature, subject to the constraints imposing inextensibility and the area A enclosed by 𝒞 (Materials and Methods). This is a well-known mechanical problem [50, 51]: The observed shape (of lowest energy) of an elastic ring enclosing a prescribed area is symmetric; higher modes of higher energy have higher numbers of lobes (Fig. 2A).

Our minimal model therefore needs one more constraint: The points at which the ring intersects the mid-sagittal cross section of the embryo cannot move freely, but their position is set at each timepoint by the progress of germ band extension. In the model, this fixes the distance d between two diametrically opposite points on the ring, i.e., its anteroposterior (AP) diameter. The shape of the deformed ring minimizes its bending energy subject to these three constraints. The corresponding Euler–Lagrange equation is

(2a) $$\kappa^{\prime \prime }(s)+\frac{\kappa {\left(s\right)}^{3}}{2}-\lambda_{0}\kappa (s)+p=0,$$

where dashes denote differentiation with respect to s, and where $\lambda_{0}$ and p are constants to be determined (Materials and Methods). We complement this with the differential equations

(2b) $$\theta^{\prime }(s)=\kappa (s),\;x^{\prime }(s)=\text{cos}\;\theta (s),\;y^{\prime }(s)=\text{sin}\;\theta (s),$$

for the tangent angle θ(s) with the AP axis, and the position (x(s),y(s)) of a point on the ring (Fig. 2B). The boundary conditions fix the enclosed area to A and the AP diameter to d and impose the symmetry of the half-ring (Fig. 2B). They are (Materials and Methods)

(3a) $$\theta (0)=-\theta (1)=\frac{\pi }{2},\;x(0)=y(0)=y(1)=0,\;x(1)=d,$$

and

(3b) $$\int_{0}^{1}[y(s)\;\text{cos}\;\theta (s)-x(s)\;\text{sin}\;\theta (s)]\;\text{d}s=A.$$

We solve this boundary-value problem numerically (Materials and Methods) as A is reduced $d=d_{0}$, the initial diameter of the ring. The lowest-energy shapes are now asymmetric about the y-axis, i.e., AP asymmetric (Fig. 2B). There are four shapes of equal energy, which include “keyhole” shapes reminiscent of the shape of the hindgut primordium. There are also AP symmetric shapes, but they have higher energy (Fig. 2B). More generally, d and A both vary as the germ band extends and the midgut invaginates. For inextensible deformations, part of (d, A) space is geometrically excluded. The asymmetric shapes remain the lowest-energy shapes in a large part of the remaining (d, A) space (Fig. 2C). This shows that the symmetry-breaking that can lead to triangular shapes is robust.

### Selection of hindgut shape by embryonic curvature

The four-fold degeneracy of the shapes of minimal energy in Fig. 2B raises the question: How does the embryo consistently select one of these orientations? To answer this, we extended our model of a planar ring to a spherical or ellipsoidal surface approximating the embryonic geometry. However, even for these simple curved surfaces, the equation analogous to Eq. (2a) becomes too complex to write down. Instead, we directly minimized the bending energy in Eq. (1), subject to the same constraints, for shapes approximated by a few Fourier terms (Materials and Methods). An elastic ring on a sphere (Fig. 2D) or at the posterior pole of an ellipsoid (Fig. 2E) still breaks symmetry as A is reduced, but the shape degeneracy persists by symmetry. If, however, the ring translates off the posterior pole and onto one side of the ellipsoid (similarly to the translation of the hindgut primordium onto the dorsal side of the embryo due to germ band extension), then the curvature gradients eliminate the degeneracy and the ring selects a triangular shape in the same orientation as the shape of the hindgut primordium (Fig. 2F).

Our minimal model thus shows that uniform contraction, representing midgut invagination, is sufficient to explain the symmetry-breaking of the hindgut primordium, with the observed shape selected by the curvature of the embryonic surface. In particular, neither active deformations of the hindgut primordium, nor inhomogeneous forces from the extending germ band that surrounds it, nor heterogeneities in its passive mechanical properties are necessary to explain the triangular shape qualitatively.

### Real-time kinematics inferred from live imaging

**FIG. 3.**
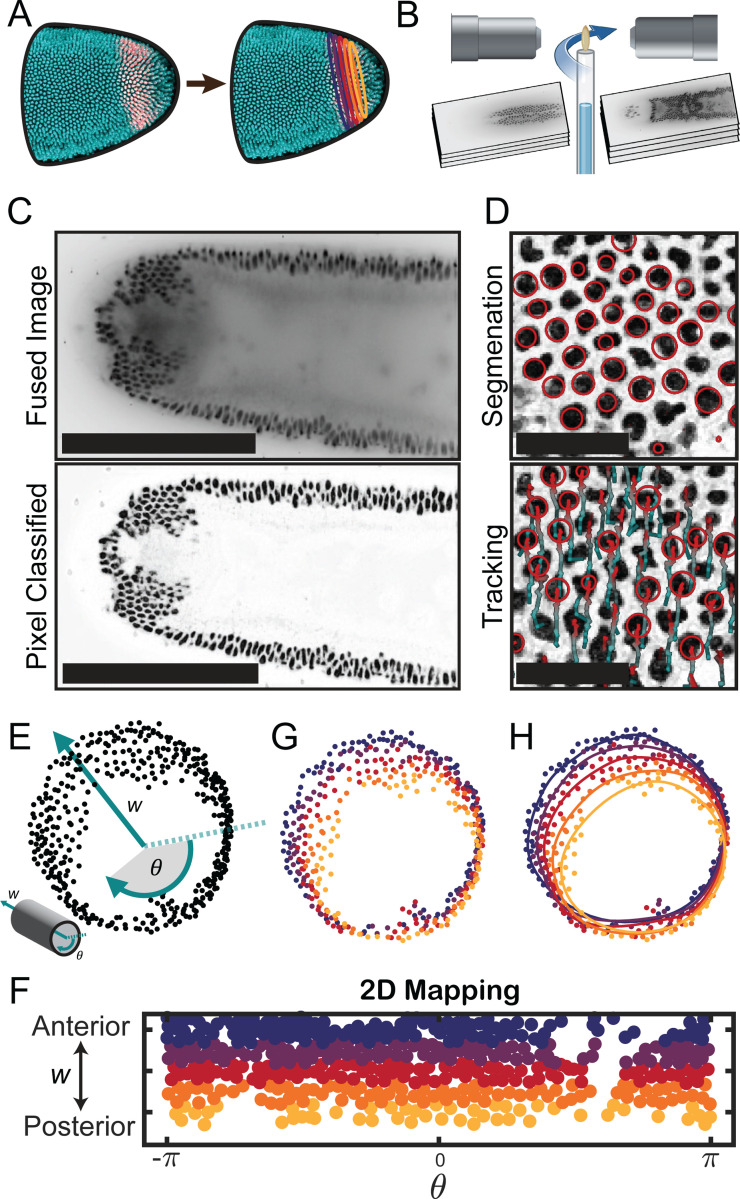
Data analysis pipeline. (A) The analysis constructs a set of closed space curves (“contours”) that are initialized by positions of nuclei within the hindgut primordium and deform with it in time. (B) Light sheet microscopy enables simultaneous imaging of both sides of embryos with fluorescent reporters for nuclei and hindgut. (C) After image fusion and deconvolution (Materials and Methods), images are processed using a pixel classifier (ilastik, 52) to improve nuclear detection. Scale bars: 200μm. (D) Nuclei within the hindgut primordium are segmented into spots (top); these spots are tracked semi-automatically using Mastodon [53] (bottom) to generate a full track for each nucleus in the hindgut primordium. Scale bars: 20μm. (E) Initial positions of nuclei at the blastoderm stage are mapped into cylindrical axial and angular coordinates w, θ (inset). (F) Nuclei are binned into contours by their anteroposterior position w in this 2D mapping. (G) Initial nuclear positions from panel (E) colored by the contour to which they are assigned from the binning in panel (F). (H) Contours are fitted using a sequence of splines that update at each timepoint as the nuclei move. Here, the initial contours are overlaid on from panel (G).

To understand the kinematics of the hindgut primordium, we generated a series of closed space curves that we term “contours”. Contours track the movement of nuclei within the hindgut primordium during the first 20 minutes of gastrulation (Fig. 3A) and visualize the deformations of the hindgut primordium as threads on the surface of a fluid visualize its flow. First, we used light sheet microscopy to image (Materials and Methods) the deforming hindgut (Fig. 3B). We cooled the embryos to slow development, increasing the effective temporal resolution, and generated a 4D dataset with isotropic spatial resolution in one or two channels at a time resolution of 6–10s. After fusing and deconvolving images, we classified pixels using a standard tool [52] to remove fluorescence from the yolk and beads used to register the images (Fig. 3C and Materials and Methods). Pixel-classified images were segmented using a difference-of-Gaussians detector that approximates nuclei as 3D spheres (54, 55, Fig. 3D). We tracked nuclei semi-automatically in Mastodon [53], a tool built on the TrackMate [54, 55] plugin for Fiji [56]. Each nuclear track was manually verified or corrected, resulting in approximately 500 tracks over approximately 100 timepoints (Fig. 3D). We initialized contours by mapping the initial nuclear positions at the blastoderm stage into cylindrical coordinates (Fig. 3E). Nuclei were binned into five groups based on their cylindrical axial coordinate w, corresponding to their embryonic anteroposterior positions (Fig. 3F). Doing so divides the ring of the hindgut primordium into five slices (Fig. 3G). Contours were fitted to each of these slices using a series of splines (Fig. 3H). Contours were continually refitted using bins propagated from the initial assignments to capture the updated nuclear positions at subsequent timepoints (Materials and Methods), revealing the kinematics of the developing hindgut (Movie S1).

### Two stages of hindgut morphogenesis

**FIG. 4.**
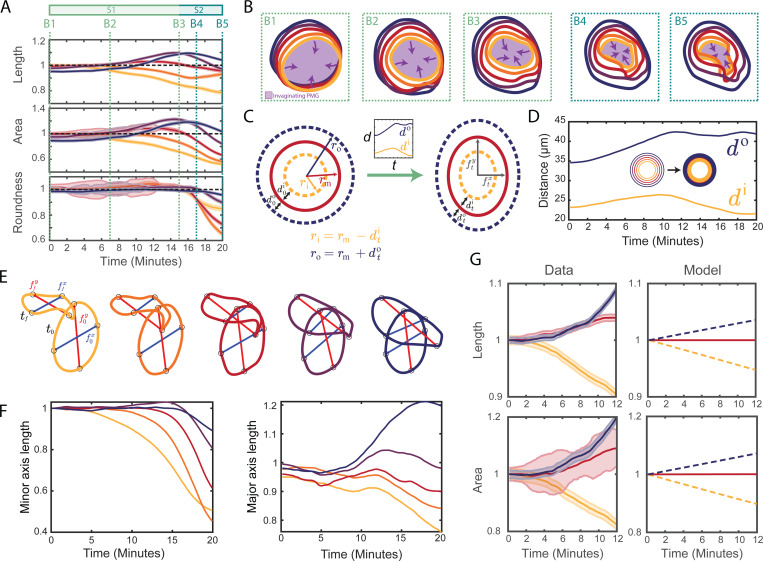
The hindgut primordium deforms in two stages. (A) Shape metrics (contour length, enclosed area, and roundness) plotted against time for a representative embryo, colored by contour (innermost, yellow to outermost, blue). There are two stages: during stage S1 (green), all contours maintain their initial roundness and the lengths and areas of the inner and outer contours decrease and increase, respectively. From t=15 min onwards (stage S2, turqoise), the areas enclosed by all contours decrease and the roundness of all contours but the outermost one decreases sharply. Dashed lines, colored by stage, indicate timepoints B1–B5 used in panel (B). Error bars are determined from the standard deviation of a simulated error distribution (Materials and Methods). (B) Contour shapes at the timepoints B1–B5 highlighted in panel (A). The violet shading indicates the invaginating posterior midgut (PMG). (C) “Coupled-ring” model of the deformation of circular contours into ellipses (Materials and Methods). At time t, the middle contour has semi-minor axis $f_{t}^{x}$ and semi-major axis $f_{t}^{y}$, and the initial distances $d_{0}^{\text{i}}$, $d_{0}^{\text{o}}$ from the middle to the inner and outer contours have changed to $d_{t}^{\text{i}}$, $d_{t}^{\text{o}}$ respectively (inset). (D) Plot of the measured mean distances $d^{\text{i}}$, $d^{\text{o}}$ from the middle to the innermost and outermost contours (Materials and Methods) against time. Inset: the contours define inner and outer rings used for calculating $d^{\text{i}}$, $d^{\text{o}}$. (E) Definition (Materials and Methods) of the minor (blue) and major (red) axis lengths $f_{t}^{x}$, $f_{t}^{y}$, shown for each contour at the initial and final timepoints $t_{0}$, $t_{f}$. (F) Plots of the minor and major axis lengths or each contour, normalized by their initial lengths, against time. (G) The “coupled-ring” model (right, Materials and Methods) sketched in panel (C) explains the kinematic behaviour of the inner and outer contours during stage S1 (left): If the length (top) or area (bottom) of the middle contour is constant (solid line), the model predicts (dashed lines) that the lengths or areas of the inner and outer contours decrease and increase, respectively, consistently with the data (left).

To quantify the contour kinematics, we computed shape metrics at each timepoint and plot the normalized length, area, and roundness of each contour in Fig. 4A for a representative embryo. The length of the middle contour changes minimally over the first twenty minutes of gastrulation, which is consistent with the approximation of an inextensible midline and use of an elastic description (as opposed to an viscous description permitting cell rearrangements) in our physical model of the symmetry-breaking. Moreover, this quantification reveals that the deformation has two stages (Fig. 4A).

During the first stage, the area and length of the outer and inner contours increase and decrease monotonically, respectively, while the area enclosed by the middle contour displays little to no change. The roundness of each contour remains close to unity, indicating uniform dilation and compression of the contours. Qualitatively, the shapes of all contours remain elliptical and begin to rotate and translate along the surface of the embryo as gastrulation begins (Figs. 4B1–4B3). Towards the end of the first stage, apical constriction of the posterior midgut causes the areas enclosed by the contours to begin to decrease, starting with the innermost contour adjacent to the posterior midgut.

The second stage involves a sharp decrease of the roundness of all contours, with the outer contours remaining rounder than the middle and inner contours (Fig. 4A). As the contours move up and around the posterior pole (Figs. 4B4 and 4B5), the midgut fully involutes and inverts, causing the areas enclosed by each contour to decrease (Fig. 4A). The contour lengths display more complex behavior, likely due to the out-of-plane deformations of the deforming hindgut. Interestingly, the inner contours, initially closer to the posterior, start to decrease in length, area, and roundness slightly before the outer contours. We computed the same metrics in terms of the position of the ring along the embryonic surface (Supplementary Fig. S5A), observing that all three shape metrics start to decrease when the ventralmost point of the contour passes the posterior pole (Supplementary Fig. S5B). This suggests that the delay results from different contours occupying similar regions of the embryo at slightly different times.

### Minimal geometric model of the observed contour kinematics

To explain the contrasting changes in the lengths and are as of the inner and outer contours during the first stage qualitatively, we introduced a minimal “coupled–ring” model describing an inner, middle, and outer contour (Fig. 4C, Materials and Methods). We hypothesized that the changes of the inner and outer contours are a consequence of the smaller deformations of the middle contour (which becomes slightly elliptical) and of the changes of its distance to the inner and outer contours. We therefore quantified (Materials and Methods) the mean distance between contours (Fig. 4D) and their major (anteroposterior) and minor (left/right) axis lengths (Figs. 4E and 4F). To explain the relative behaviors of the inner and outer contours, we first modeled the length of the middle contour to be constant because of its lesser length change during stage S1. This predicts that the lengths of the inner and outer contours decrease and increase, respectively (Fig. 4G). Similarly, assuming that the area of the middle contour is constant, the model shows a decrease and increase of the inner and outer contour areas, respectively (Fig. 4G). The “coupled-ring” model thus captures the observed kinematics of the innermost and outermost contours.

Interestingly, the major axis (i.e., the anteroposterior diameter) of the outermost contour has lengthened significantly by the end of the process (Fig. 4F), while the major axes of the other contours remain constant or shorten. At the same time, the shape of the outermost contour remains roundest (Fig. 4A). Only the middle and inner contours adopt the triangular shape that we have predicted in our minimal planar model. This is consistent with our model because shapes do not break symmetry if their anteroposterior diameter increases too much, as is the case for the blue outermost contour (Fig. 2C).

## III. DISCUSSION

Any developmental system comprised of both actively-deforming and passive tissues [30, 31, 34, 35, 37, 38] inevitably features deformations in boundary regions bridging actively-deforming neighbors. Such “boundary-driven morphogenesis” has proven difficult to understand, even at the level of kinematics, due to complex combinations of in-plane and out-of-plane deformations. This difficulty is compounded by the facts that boundary-driven and active morphogenesis can combine within the same tissue and that different combinations of passive and active cell behaviors can generate similar tissue deformations [40, 41, 47]. We have shown that our understanding of the morphogenesis of the *Drosophila* hindgut primordium is consistent with a minimal model in which its complex deformations result solely from the forces exerted by its actively deforming neighboring tissues and the ellipsoidal geometry of the eggshell. Its dramatic change in shape, well-characterized neighboring tissues, and compatibility with well-established techniques for *Drosophila* cell biology make the hindgut an ideal model for boundary-driven morphogenesis.

Previous work has described specific cellular processes in embryonic primordia ranging from the internalization of cells in the mesoderm [48, 57–59] to biased cell rearrangements in the germ band [41, 43, 47], proposing critical insights into how deformations may occur. Ultimately, fully understanding morphogenesis requires a more global approach that can integrate these individual findings. Here, we have taken such an approach that has allowed us to examine the full deformation of the hindgut primordium in its biological context. Our mechanism depends only on a uniform reduction of apical area by invagination of the posterior midgut and a uniform boundary condition from the germ band that translates the ring off the posterior pole. Movement of the ring to a region where the eggshell imposes anisotropic embryonic curvatures resolves the degeneracy of this symmetry-breaking and selects a triangular shape with proper orientation. Our minimal model absorbs these complex in-plane and out-of-plane deformations into simplified yet biologically relevant and measurable parameters, including the area enclosed by the tissue and its anteroposterior diameter. This paradigm will also be able to resolve which physical effects are likely to drive the observed global morphological changes in other developmental processes with complex boundary conditions.

Although we have distilled the complex 3D shape of the hindgut that we observed in Fig. 1D into a triangular shape on the surface of the embryo, future work will need to understand the out-of-plane deformations of the internalized “keyhole” shape where the propagating ventral furrow meets the involuted midgut (Fig. 1D). In addition, we observed some in-plane stretching of the tissue between the contours in the anteroposterior direction, as evidenced by the changing intercontour distances (Fig. 4D). Further work will need to resolve the mechanical basis for this deformation within the hindgut. Continuum mechanical approaches [60] will enable elucidating the contributions of in-plane and out-of-plane boundary conditions from the neighboring active tissues to these and other characteristics of the hindgut shape. This will be aided by the rapid advances in techniques for measuring passive tissue properties [61–66], perturbing cytoskeletal elements [67, 68], and machine-learning-assisted computer vision [69–72], all of which will ultimately be used to populate a descriptive atlas of morphogenesis [73]. This approaching wave of data will couple to our framework to resolve mechanisms for global morphologies in development.

More generally, by demonstrating the possible role of embryonic curvature in selecting the orientation of the triangular shape of the hindgut primordium, our work also offers an explanation for the effect of embryonic geometric constraints on the morphogenesis of other tissues. In many organisms, Brachyury is expressed at the lip of the blastopore or a similar invaginating structure [19, 22, 24, 74] that deforms into various shapes depending on the organism. In some of these organisms, the blastopore lip appears as a constricting ring on a spherical embryo that fluidizes through cell rearrangements or oriented divisions, which can relieve stresses imposed at the boundaries through internal viscous dissipation [75–78]. In some insects with more elongated embryos than those of *Drosophila*, such as the medfly, germ band extension and posterior invagination differ, yet the lip of the posterior invagination also looks triangular as it moves off the posterior pole [79]. In the beetle *Tribolium castaneum*, the serosa undergoes epiboly through a mechanism separate from germ band extension and forms an intermediate triangular window on the ventral side of the ellipsoidal embryo [80, 81]. Using our framework to understand the mechanisms that drive the emergence of blastopore shapes will provide further insights into the evolution of the blastopore-to-primitive streak transition [82, 83].

More physically, our triangular shape bifurcation expands the large body of work on constrained elastic lines in the plane and on curved surfaces [50, 51, 84–91] and related problems [92–95]. In this context, the shape-selection mechanism that we propose stresses the importance of anisotropic curvature for such bifurcations. The hindgut primordium and the ellipsoidal *Drosophila* embryo more generally therefore provide a paradigm for mechanical bifurcations *within* curved surfaces. Indeed, very recent work has shown that even the minimal instability that is Euler buckling changes fundamentally within general curved surfaces [96], but, compared to the well-understood instabilities *of* curved surfaces [30, 97–104], these instabilities within curved surfaces remain mysterious.

## Supplementary Material

Supplement 1
