# Supplementary material for: A model for boundary-driven tissue morphogenesis
Source: ArXiv. 2025 Mar 5:arXiv:2503.03688v1. Preprint. [Version 1] (PMC11908361)
Supplement: Supplement 1 [file NIHPP2503.03688v1-supplement-1.pdf]

APPENDIX A. MATERIALS & METHODS

1. Imaging and tracking

In order to visualize nuclei and identify hindgut progenitors, we generated a line containing the histone tag Histone H2B-RFP with the maternal ubiquitously-expressed GFP under the *bicoid* promoter. Females from this stock were crossed with males containing the previously-generated Brachyenteron LlamaTag [7, 49]. To generate the movie stills showing lateral and dorsal views of the deforming hindgut, embryos were manually dechorionated on double-sided tape before being immersed in halocarbon oil on custom filter slides and imaged using a Leica SP5 scanning confocal microscope. For tracking, embryos were manually dechorionated on double-sided tape and mounted in capillary tubes containing a solution of 1% agarose with 1:200 diluted TetraSpeck 0.2 µm microspheres (ThermoFisher #T7280). Imaging was performed on a Bruker/Luxendo MuVi-SPIM light-sheet microscope at 33.3× magnification using two cameras mounted opposite each other and a rotating stage (Fig. 3B). Syncytial embryos were cooled to 18 °C and full stacks were taken in the sagittal and frontal planes and in two channels (nuclei and Byn reporter) every 60 seconds to monitor the progression of development and designate cell identities. At the onset of gastrulation, defined as the onset of ventral furrow formation, imaging was switched to a single image stack acquired through the frontal plane (through the dorsoventral axis) in the histone reporter channel every 7.75 seconds at slice thicknesses of 1 µm to maximize temporal resolution. After 12–18 minutes, the imaging mode was switched back to the initial 2-channel, 2-angle mode to monitor further development. Embryos with visibly aberrant development or arrest were discarded from the dataset. Nuclei were tracked using Mastodon (53 and *Materials and Methods*).

2. Construction of contours from data

Raw tracks were smoothed by using an exponential moving average filter on each spatial dimension with a window size of 10 timepoints, or 80–110 seconds. Only nuclei that could be tracked through each timepoint were used. Approximately 5–10% of nuclei, typically contained within the ventral midline in the ventral furrow, could not be tracked reliably throughout the full movie. To initialize contours, positions of nuclei at the first timepoint were mapped into cylindrical coordinates (Fig. 3E). Positions were first normalized and then projected into eigenspace using a correlation matrix. Coordinates in eigenspace were converted to cylindrical coordinates, of which only the polar angle and the axial coordinate were used for mapping. Nuclei were binned into 5 bins based on their axial coordinates, corresponding to bands 2–3 nuclei wide to be used to fit contours. Each bin defined an initial contour identity, and these were propagated forward in time as nuclear positions changed.

To generate a contour at a given timepoint, points within the corresponding bin were sorted based on their initial azimuthal angle and their updated spatial coordinates were repeated three times to reduce edge effects. A cubic smoothing spline was applied to each dimension using the `csaps` function in MATLAB (The MathWorks, Inc.) with a smoothing parameter of 0.01. To extract a single closed contour, we iterated simultaneously in the forward and backward directions from the midpoint of the repeated array that contains the knots of these splines until these knots fell within a fixed tolerance of each other (which indicates completion of a full loop). A closed space curve was then obtained by joining the two knots and discarding knots outside of the interval containing the midpoint. All contours were resampled to generate space curves of 500 knots with constant arclength spacing.

3. Calculation of shape metrics

The length L of a contour was computed as the sum of the arc lengths of each spline within that contour. Its area A was calculated by identifying its dorsalmost and ventralmost points to creating a line of bilateral symmetry. From this, the area was obtained as the (Riemann) sum of the lengths of line segments between corresponding points on either side of this midline multiplied by the distance between them. The roundness was defined to be $R = A/L^2$. In Fig. 4, each of these metrics was normalized by its value when the ventralmost point of the respective contour was located at a reference position. For this purpose, we first approximated the surface of the embryo by an ellipsoid with aspect ratio 2.5 : 1 : 1, based on the aspect ratio 185 µm : 92.5 µm : 92.5 µm of the representative embryo used for Fig. 4 measured using Fiji [56]. This reference position was then chosen to be the initial position of ventralmost point of the innermost (yellow) contour. To obtain metrics in terms of the positions of the contours (Fig. S5), we reparameterized contours similarly by the positions of their ventralmost points along the arclength of a sagittal cross section of this ellipsoid, $s(\theta) = \mathrm{E}(\theta, \varepsilon)$, where $\mathrm{E}(\theta, \varepsilon)$ is the incomplete elliptic integral of the second kind, $\varepsilon = 0.92$ is the eccentricity of this elliptical cross section, and θ is the polar angle measured from the anteroposterior axis.

4. Physical models

Details of the derivation of the physical models are given in Appendix B.

5. Additional experimental and image analysis methods

Further details of the experimental and image analysis methods are given in Appendix C.

APPENDIX B. PHYSICAL MODELS

1. Derivation of the equation governing an inextensible elastic ring in the plane

As discussed in the main text, we begin by modeling the primordial hindgut as an inextensible elastic ring of length $\ell = 2$ in the plane (Fig. S1A), which we endow with Cartesian coordinates (x, y). We parameterize the ring by its arclength s, so that a point on it has position $\boldsymbol{r}(s) = \bigl(x(s), y(s)\bigr)$. It is useful to consider half of the ring in the subsequent calculations and thus restrict to $s \in [0, 1]$ (Fig. S1A). The deformed shape of the ring minimizes its bending energy subject to two constraints: (1) local inextensibility, $\|\boldsymbol{r}'(s)\| = 1$, where the dash denotes differentiation with respect to s, and (2) the constraint that the half-ring enclose an area $A/2$, associated with midgut invagination, as discussed in the main text. The Lagrangian of the problem is

$$\mathcal{L} = \frac{1}{2}\int_0^1 \|\boldsymbol{r}''(s)\|^2\,\mathrm{d}s + \int_0^1 \lambda(s)\left[\|\boldsymbol{r}'(s)\|^2 - 1\right]\mathrm{d}s + p\left(\int_0^1 \frac{\boldsymbol{r}(s)\cdot\boldsymbol{n}(s)}{2}\,\mathrm{d}s - \frac{A}{2}\right), \tag{B1}$$

in which the first term is the bending energy, with $\|\boldsymbol{r}''(s)\|^2 = \kappa(s)^2$, the squared curvature of the elastic line. In the other terms, $\lambda(s)$ is the Lagrange multiplier function associated with the inextensibility constraint, p is the Lagrange multiplier that enforces area conservation, and $\boldsymbol{n}(s)$ is the unit normal to the ring. The tangent $\boldsymbol{t}(s) = \boldsymbol{r}'(s)$ and $\boldsymbol{n}(s)$ obey $\boldsymbol{t}'(s) = \kappa(s)\boldsymbol{n}(s)$ and $\boldsymbol{n}'(s) = -\kappa(s)\boldsymbol{t}(s)$.

To be able to calculate the variation of the third term in Eq. (B1), we need to note that $\boldsymbol{n}(s)\cdot\boldsymbol{n}(s) = 1 \Longrightarrow \delta\boldsymbol{n}(s)\cdot\boldsymbol{n}(s) = 0$ and $\boldsymbol{n}(s)\cdot\boldsymbol{t}(s) = 0 \Longrightarrow \delta\boldsymbol{n}(s)\cdot\boldsymbol{t}(s) = -\delta\boldsymbol{t}(s)\cdot\boldsymbol{n}(s)$, which imply $\delta\boldsymbol{n}(s) = -\left[\delta\boldsymbol{t}(s)\cdot\boldsymbol{n}(s)\right]\boldsymbol{t}(s)$. With this, and on integrating by parts several times, we obtain

$$\begin{aligned}\delta\mathcal{L} = \int_0^1 \Biggl\{\Biggl[\boldsymbol{t}'''(s) + 2\lambda(s)\boldsymbol{t}'(s) + 2\lambda'(s)\boldsymbol{t}(s) + \frac{p}{2}\boldsymbol{n}(s) + \frac{p}{2}\frac{\mathrm{d}}{\mathrm{d}s}\Bigl(\left[\boldsymbol{r}(s)\cdot\boldsymbol{t}(s)\right]\boldsymbol{n}(s)\Bigr)\Biggr]\cdot\delta\boldsymbol{r}(s) + \delta\lambda(s)\left[\boldsymbol{r}'(s)\cdot\boldsymbol{r}'(s) - 1\right]\Biggr\}\mathrm{d}s \\ + \left[\!\left[\boldsymbol{r}''(s)\cdot\delta\boldsymbol{r}'(s) + \Bigl(2\lambda(s)\boldsymbol{t}(s) - \boldsymbol{t}''(s) - \frac{p}{2}\left[\boldsymbol{r}(s)\cdot\boldsymbol{t}(s)\right]\boldsymbol{n}(s)\Bigr)\cdot\delta\boldsymbol{r}(s)\right]\!\right]_0^1.\end{aligned} \tag{B2}$$

Hence

$$\boldsymbol{t}'''(s) + 2\lambda(s)\boldsymbol{t}'(s) + 2\lambda'(s)\boldsymbol{t}(s) + p\boldsymbol{n}(s) + \frac{p}{2}\kappa(s)\left[\boldsymbol{r}(s)\cdot\boldsymbol{n}(s)\right]\boldsymbol{n}(s) - \frac{p}{2}\kappa(s)\left[\boldsymbol{r}(s)\cdot\boldsymbol{t}(s)\right]\boldsymbol{t}(s) = \boldsymbol{0}. \tag{B3}$$

Differentiating $\boldsymbol{t}'(s) = \kappa(s)\boldsymbol{n}(s)$ gives $\boldsymbol{t}''(s) = \kappa'(s)\boldsymbol{n}(s) - \kappa(s)^2\boldsymbol{t}(s)$ and $\boldsymbol{t}'''(s) = \kappa''(s)\boldsymbol{n}(s) - 3\kappa(s)\kappa'(s)\boldsymbol{t}(s) - \kappa(s)^3\boldsymbol{n}(s)$. With this, the normal and tangential components of Eq. (B3) yield, respectively,

$$\kappa''(s) - \kappa(s)^3 - 2\lambda(s)\kappa(s) + p + \frac{p}{2}\kappa(s)\left[\boldsymbol{r}(s)\cdot\boldsymbol{n}(s)\right] = 0, \qquad -3\kappa(s)\kappa'(s) + 2\lambda'(s) - \frac{p}{2}\kappa(s)\left[\boldsymbol{r}(s)\cdot\boldsymbol{t}(s)\right] = 0, \tag{B4}$$

whence we obtain expressions for $\boldsymbol{r}(s)\cdot\boldsymbol{n}(s)$, $\boldsymbol{r}(s)\cdot\boldsymbol{t}(s)$, which, in turn, give

$$\boldsymbol{r}(s) = \frac{2}{p\kappa(s)}\left\{\left[\kappa''(s) - \kappa(s)^3 + 2\lambda(s)\kappa(s) + p\right]\boldsymbol{n}(s) + \left[-3\kappa(s)\kappa'(s) + 2\lambda'(s)\right]\boldsymbol{t}(s)\right\}. \tag{B5}$$

We can now differentiate this relation, use $\boldsymbol{r}'(s) = \boldsymbol{t}(s)$, and collect normal and tangential components to find

$$\kappa'''(s)\kappa(s) - \kappa'(s)\kappa''(s) + \kappa'(s)\kappa(s)^3 - p\kappa'(s) = 0, \qquad \kappa''(s) + \frac{\kappa(s)^3}{2} - \lambda(s)\kappa(s) - \frac{p}{4} - \frac{\mathrm{d}}{\mathrm{d}s}\left(\frac{\lambda'(s)}{\kappa(s)}\right) = 0. \tag{B6}$$

The second equation gives the Lagrange multiplier function $\lambda(s)$ once $\kappa(s)$ is determined from the first, which rearranges to

$$\frac{\mathrm{d}}{\mathrm{d}s}\left(\frac{\kappa''(s)}{\kappa(s)}\right) + \kappa(s)\kappa'(s) + p\frac{\mathrm{d}}{\mathrm{d}s}\left(\frac{1}{\kappa(s)}\right) = 0. \tag{B7}$$

On integrating, we finally obtain

$$\kappa''(s) + \frac{\kappa(s)^3}{2} - \lambda_0\kappa(s) + p = 0, \tag{B8}$$

where λ_0 is a constant of integration. [In deriving this equation, we have assumed that $\kappa(s) \neq 0$, but, if $\kappa(s) = 0$, then the final equation still holds by the first of Eqs. (B4).] We are not aware of a previous derivation of this governing equation by direct variation of the Lagrangian, but the same equation has been obtained using the method of "normal variation" [105, 106] or from the Kirchhoff rod equations [107, 108].

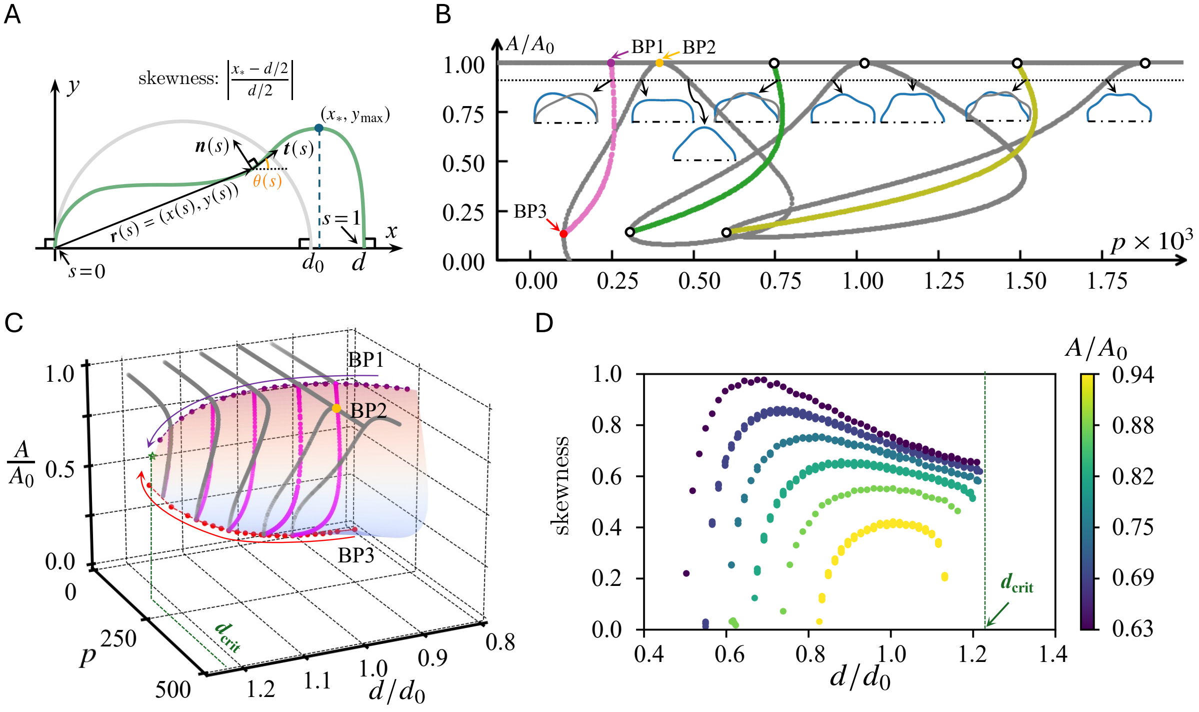

FIG. S1. Elastic ring in the plane: Bifurcation diagrams. (A) Deformation of an inextensible elastic half-ring of length $\ell/2 = 1$ in Cartesian axes (x, y). A circular half-ring (gray line) encloses an area $A_0 \equiv 1/\pi$ and has diameter $d_0 \equiv 2/\pi$. The ring deforms as the area A enclosed by the ring and its diameter d are varied while minimizing its bending energy. The position of a point on the deformed half-ring (green line) is $\boldsymbol{r}(s) = (x(s), y(s))$, where $s \in [0, 1]$ is arclength, so that $x(1) - x(0) = d, y(0) = y(1)$. The tangent and normal to the ring are $\boldsymbol{t}(s)$ and $\boldsymbol{n}(s)$, and the tangent angle is $\theta(s)$. Completing the shape of the half-ring into a full ring requires $\theta(0) = \pi/2, \theta(1) = -\pi/2$ (right angles emphasized). Inset equation: definition of the skewness of the deformed shape. (B) Bifurcation diagram of the elastic half-ring for $d = d_0$ in (p, A) space, where p is pressure. Symmetric branches (gray lines) and asymmetric branches (colored lines) bifurcate off the undeformed branch $A = A_0$ at increasing values of p. Each asymmetric branch joins a symmetric branch. Circular markers are branch points; the first three branch points BP1, BP2, BP3 are highlighted. Insets: deformed shapes for $A/A_0 = 0.94$ on different branches; on asymmetric branches, two shapes reflected about $s = 1/2$ are shown to emphasize the asymmetry. The first branch to bifurcate (magenta branch) is an asymmetric keyhole shape (similar to the shape of the posterior hindgut). (C) Plot of the first asymmetric branch (shaded surface) in (d, p, A) space. Magenta lines are sections at constant d; grey lines are the corresponding symmetric branches linked to these by the branch points BP1 and BP3. These points merge as $d \longrightarrow d_{crit} \approx 1.22$; the first asymmetric branch ceases to exist for $d > d_{crit}$. The symmetric branches break up and BP2 disappears for $d \neq d_0$. (D) Plot of the skewness of the first asymmetric branch against d for different A.

To write down the boundary conditions, we introduce the tangent angle $\theta(s)$ (Fig. S1A). Since $\theta'(s) = \kappa(s)$, Eq. (B8) is a third-order equation for $\theta(s)$. By definition, $x'(s) = \cos\theta(s)$, $y'(s) = \sin\theta(s)$, which are two first-order equations. Moreover, we are to determine two unknown constants, viz., λ_0, p. We therefore need $3 + 1 + 1 + 2 = 7$ boundary conditions. We impose (Fig. S1A)

$$\theta(0) = \frac{\pi}{2}, \quad \theta(1) = -\frac{\pi}{2}, \quad x(0) = 0, \quad x(1) = d, \quad y(0) = y(1) = 0, \quad \int_0^1 \left[y(s)\cos\theta(s) - x(s)\sin\theta(s)\right] \mathrm{d}s = A, \tag{B9}$$

in which d is the anterior-posterior extension of the ring (set by germband extension, as discussed in the main text), and the first two conditions ensure that the full ring does not have kinks at $s = 0$ or $s = 1$ (Fig. S1A). With these boundary conditions, the boundary terms in Eq. (B2) vanish as required, because they imply $\delta\boldsymbol{r}(0) = \delta\boldsymbol{r}(1) = \delta\boldsymbol{r}'(0) = \delta\boldsymbol{r}'(1) = \boldsymbol{0}$.

2. Bifurcation diagrams

To study how a circular ring ($A = A_0 \equiv 1/\pi, d = d_0 \equiv 2/\pi$) deforms as A and d vary, we solve these equations using `AUTO-07p` [109]. We first consider the case $d = d_0$, and plot the bifurcation diagram in (p, A) space (Fig. S1B). The first branch to bifurcate off the undeformed shape $A = A_0$, at branch point BP1, has asymmetric keyhole solutions akin to the shape of the hindgut (Fig. S1B, inset, as discussed in the main text). The first symmetric shapes only bifurcate at higher p, at branch point BP2. The asymmetric branch ends at another branch point, BP3, where it connects to this symmetric branch. As p increases, additional symmetric and asymmetric branches bifurcate off $A = A_0$, with the asymmetric branches connecting to the symmetric branches that snake around them (Fig. S1B).

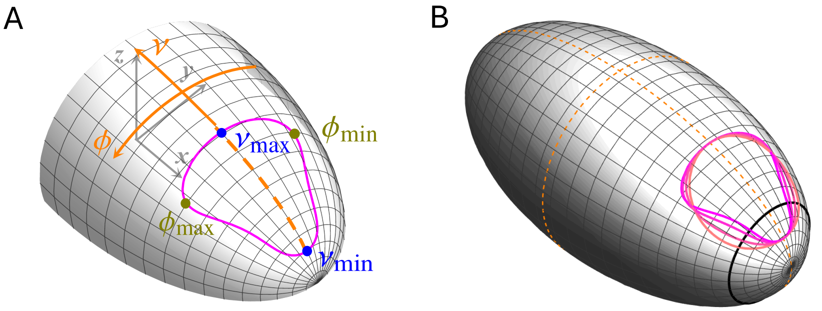

FIG. S2. Extended model: Elastic ring on a curved surface. (A) Geometry of a closed curve Γ_Ω on an ellipsoid with semi-axes $a, b = c$, in terms of the polar angles ν, ϕ. The position of the curve is defined by anterior-most and posterior-most points $\phi = 0$, $\nu = \nu_{\text{min}}, \nu_{\text{max}}$, and the curve encloses the area $\phi_{\text{min}}(\nu) \leqslant \phi \leqslant \phi_{\text{max}}(\nu)$ for $\nu_{\text{min}} \leqslant \nu \leqslant \nu_{\text{max}}$. (B) Example result of the extended model: An elastic circular ring moved from the posterior pole (initial black ring) to the dorsal side breaks symmetry when the area A enclosed by the ring is reduced. Parameter values: $a = 2, b = c = 1$; initial circle: $\nu = 0.5$.

As d varies (Fig. S1C), BP1 moves, in (d, p, A) space, to $A < A_0$, but the asymmetric branch continues to be the lowest branch where it exists. Indeed, at $d = d_{\text{crit}} \approx 1.22$, BP1 merges with BP3, and this asymmetric branch ceases to exist. For $d \neq d_0$, branch point BP2 disappears, and the symmetric branches that merge there at $d = d_0$ break up (Fig. S1C). As discussed in the main text, this bifurcation diagram shows that the asymmetric branch is the lowest (and hence the observed branch) for a range for values around $d = d_0$.

We quantify the asymmetry of these shapes by computing the skewness of the corresponding shapes (Fig. S1A). The plot of skewness against d (Fig. S1D) emphasizes how, for each value of $A < A_0$, solutions of non-zero skewness exist in a range of values of $d < d_{\text{crit}}$.

3. Extended model: Mechanics of an inextensible elastic ring on a curved surface

As discussed in the main text, we extend our model to describe an inextensible elastic ring on a curved surface. Similarly to the plane case, the shape of a curve Γ of prescribed length and enclosed area, confined to lie on a surface Ω, is determined by minimizing the bending energy of the curve,

$$E = \frac{1}{2}\oint_\Gamma \kappa(s)^2\,\mathrm{d}s, \tag{B10}$$

where $\kappa(s)$ is the total curvature of Γ and s is its arclength, subject to the constraints of prescribed length, prescribed surface area, and the constraint of the curve lying on Ω.

We now specialize to ellipsoidal surfaces (Fig. S2A), which include spherical surfaces as a special case, and begin by imposing the condition $\Gamma \subset \Omega$ by choosing an explicit parametrization of Γ. In a Cartesian coordinate system with position vector $\boldsymbol{r} = (x, y, z)$, the position of a point on the surface of an ellipsoid can be written as

$$x(\nu) = a\cos\nu, \qquad y(\nu,\phi) = b\cos\nu\sin\phi, \qquad z(\nu,\phi) = c\cos\nu\cos\phi, \tag{B11}$$

where a, b, c are the semi-axes of the ellipsoid, and $\nu \in [0, \pi]$ and $\phi \in [0, 2\pi]$ are its polar and azimuthal angles, respectively. To impose $\Gamma \subset \Omega$, we choose a parametrization

$$\Gamma_\Omega(\tau)\colon \tau \longrightarrow \bigl\{x\bigl(\nu(\tau),\phi(\tau)\bigr), y\bigl(\nu(\tau),\phi(\tau)\bigr), z\bigl(\nu(\tau),\phi(\tau)\bigr)\bigr\}, \tag{B12}$$

where $\tau \in [0, T]$ parametrizes the curve, for some $T > 0$. The total squared curvature of Γ is now

$$\kappa(\tau)^2 = \frac{[z''(\tau)y'(\tau) - y''(\tau)z'(\tau)]^2 + [x''(\tau)z'(\tau) - z''(\tau)x'(\tau)]^2 + [x''(\tau)y'(\tau) - y''(\tau)x'(\tau)]^2}{\bigl[x'(\tau)^2 + y'(\tau)^2 + z'(\tau)^2\bigr]^{3/2}}, \tag{B13}$$

where dashes now denote differentiation with respect to τ. The length of the curve and the area of the enclosed region are

$$\oint_{\Gamma_\Omega} \mathrm{d}s = \int_0^T \bigl[x'(\tau)^2 + y'(\tau)^2 + z'(\tau)^2\bigr]^{1/2}\,\mathrm{d}\tau, \qquad \oiint_{\Gamma_\Omega} \mathrm{d}A = \int_{\nu_{\text{min}}}^{\nu_{\text{max}}}\int_{\phi_{\text{min}}(\nu)}^{\phi_{\text{max}}(\nu)} \left\| \frac{\partial \mathbf{r}}{\partial \nu} \times \frac{\partial \mathbf{r}}{\partial \phi} \right\| \mathrm{d}\phi\,\mathrm{d}\nu, \tag{B14}$$

respectively, where the values $\phi_{\text{min}}(\nu), \phi_{\text{max}}(\nu)$ and $\nu_{\text{min}}, \nu_{\text{max}}$ are associated with the shape of the curve (Fig. S2A). A curve Γ of prescribed length L and prescribed enclosed area A on Ω now extremizes the functional

$$\mathcal{F} = \oint_{\Gamma_\Omega} \kappa(\tau)^2 s'(\tau)\,\mathrm{d}\tau + \lambda_1\left(\oiint_{\Gamma_\Omega} \mathrm{d}A - A\right) + \lambda_2\left(\oint_{\Gamma_\Omega} \mathrm{d}s - L\right), \tag{B15}$$

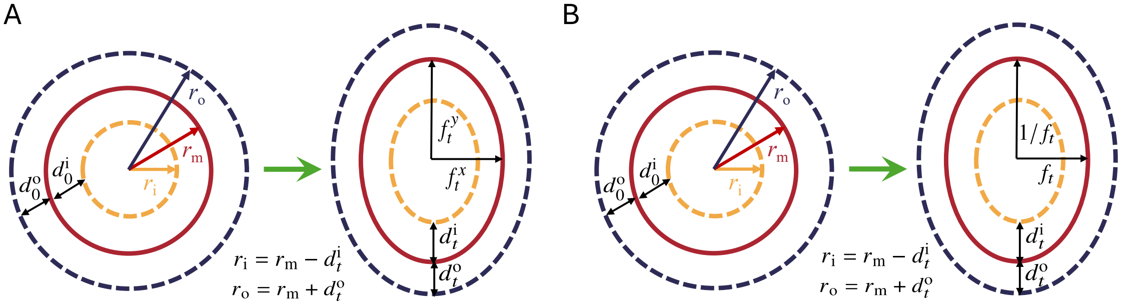

FIG. S3. "Coupled-ring" model. The model consists of three concentric circles in the plane that have initial radii $r_{\mathrm{i}}, r_{\mathrm{m}}, r_{\mathrm{o}}$, which satisfy $r_{\mathrm{o}} - r_{\mathrm{m}} = r_{\mathrm{m}} - r_{\mathrm{i}} = d_0^{\mathrm{o}} = d_0^{\mathrm{i}}$. As these rings deform into ellipses with semi-minor axis f_t^x and semi-major axis f_t^y at time t, the distances between the rings change to $d_t^{\mathrm{o}}, d_t^{\mathrm{i}}$, respectively. We analyze two cases, in which (A) the length of the middle is conserved and (B) the area of the middle ring is conserved.

where $s'(\tau) = \left[x'(\tau)^2 + y'(\tau)^2 + z'(\tau)^2\right]^{1/2}$. The first term on the right-hand side is the bending energy of the curve (up to a factor of $1/2$), and the second and third terms impose the area and length constraints by Lagrange multipliers λ_1, λ_2.

Variation of $\mathcal{F}$ with respect to the functions $\nu(\tau), \phi(\tau)$ determines, in principle, a boundary-value problem that sets the shape of the energy-minimizing curve. However, it turns out that the calculations determining this boundary-value problem are prohibitively cumbersome. We therefore take a different numerical approach: For simplicity, we consider a curve that can be expressed in terms of a few Fourier coefficients, viz.,

$$\nu(\tau) = b_0 + b_1 \cos\tau, \qquad \phi(t) = a_1 \sin\tau + a_2 \sin 2\tau + a_3 \sin 3\tau. \tag{B16}$$

In what follows, we will determine b_0, b_1 by imposing the positions $\nu_{\min}, \nu_{\max}$ of the anterior-most and posterior-most points along the curve on the ellipsoid (Fig. S2A). The length and area constraints then give two relations between the remaining three parameters a_1, a_2, a_3, and their values are finally set by minimizing the elastic bending energy subject to these relations numerically. This numerical minimization is performed using the `NMinimize` function of Mathematica (Wolfram, Inc.).

In Fig. S2B, we illustrate this approach, starting from a circle of length A_0 and circumference L_0. We move this curve along the ellipsoid to a different position on its dorsal side, and minimize $\mathcal{F}$ for $L = L_0$ and $A < A_0$. Reducing the imposed area enclosed by the curve in this way, we find a symmetry breaking leading to keyhole shapes of the hindgut, as discussed in more detail in the main text.

4. "Coupled-ring" model

We construct a model of coupled rings that deform into ellipses to explain the observed length and area changes of the inner and outer contours, discussed in the main text, qualitatively. Here we provide the details of the derivation of the model. We consider three concentric circles with initial lengths and areas

$$L_0^{\mathrm{i}} = 2\pi\left(r_{\mathrm{m}} - d_0^{\mathrm{i}}\right), \qquad L_0^{\mathrm{m}} = 2\pi r_{\mathrm{m}}, \qquad L_0^{\mathrm{o}} = 2\pi\left(r_{\mathrm{m}} + d_0^{\mathrm{o}}\right), \tag{B17a}$$

$$A_0^{\mathrm{i}} = \pi\left(r_{\mathrm{m}} - d_0^{\mathrm{i}}\right)^2, \qquad A_0^{\mathrm{m}} = \pi r_{\mathrm{m}}^2, \qquad A_0^{\mathrm{o}} = \pi\left(r_{\mathrm{m}} + d_0^{\mathrm{o}}\right)^2, \tag{B17b}$$

where sub- or superscripts i, m, o refer to the inner, middle, and outer rings, respectively and where r_{m} is the initial radius of the middle ring and $d_0^{\mathrm{i}}, d_0^{\mathrm{o}}$ denote the initial distances of the middle ring to the inner and outer rings, respectively. We assume that, as the inner rings deforms into an ellipse with semi-minor and semi-major axes f_t^x, f_t^y, respectively, the inner and outer rings deform into ellipses with axes $f_t^x - d_t^{\mathrm{i}}, f_t^y - d_t^{\mathrm{i}}$ and $f_t^x + d_t^{\mathrm{o}}, f_t^y + d_t^{\mathrm{o}}$, respectively, where $d_t^{\mathrm{i}}, d_t^{\mathrm{o}}$ are the distances between rings at time t. Hence the eccentricities of the ellipses are

$$\varepsilon_t^{\mathrm{i}} = \left[1 - \left(\frac{f_t^x - d_t^{\mathrm{i}}}{f_t^y - d_t^{\mathrm{i}}}\right)^2\right]^{1/2}, \qquad \varepsilon_t^{\mathrm{m}} = \left[1 - \left(\frac{f_t^x}{f_t^y}\right)^2\right]^{1/2}, \qquad \varepsilon_t^{\mathrm{o}} = \left[1 - \left(\frac{f_t^x + d_t^{\mathrm{o}}}{f_t^y + d_t^{\mathrm{o}}}\right)^2\right]^{1/2}. \tag{B18}$$

The lengths and areas of the rings at time t are therefore

$$L_t^{\mathrm{i}} = 4\left(f_t^y - d_t^i\right)\mathrm{E}(\varepsilon_t^{\mathrm{i}}), \qquad L_t^{\mathrm{m}} = 4 f_t^y \mathrm{E}(\varepsilon_t^{\mathrm{m}}), \qquad L_t^{\mathrm{o}} = 4\left(f_t^y + d_t^o\right)\mathrm{E}(\varepsilon_t^{\mathrm{o}}), \tag{B19a}$$

$$A_t^{\mathrm{i}} = \pi\left(f_t^y - d_t^i\right)\left(f_t^x - d_t^i\right), \qquad A_t^{\mathrm{m}} = \pi f_t^x f_t^y, \qquad A_t^{\mathrm{o}} = \pi\left(f_t^y + d_t^o\right)\left(f_t^x + d_t^o\right), \tag{B19b}$$

in which $\mathrm{E}(\varepsilon)$ is the complete elliptic integral of the second kind. We now consider two cases: In the first case, the length of the middle ring is conserved (Fig. S3A). The condition $L_t^{\mathrm{m}} = L_0^{\mathrm{m}}$ is an equation for f_t^x given f_t^y which can be solved numerically. In the second case, the area of the middle ring is conserved (Fig. S3B), and the condition $A_t^{\mathrm{m}} = A_0^{\mathrm{m}}$ yields $f_t^x = r_{\mathrm{m}}^2 / f_t^y$.

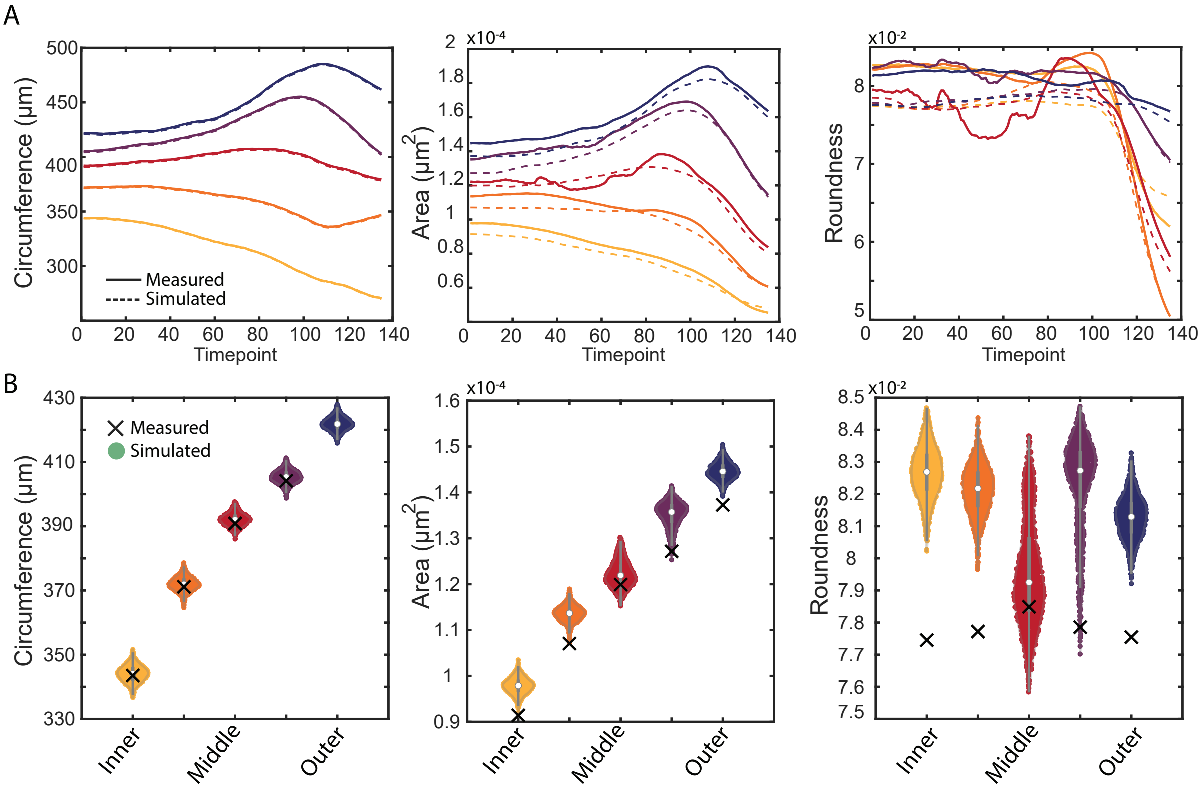

FIG. S4. Analysis of the simulated error. (A) Plots of the means of the simulated contour circumferences, areas, and roundnesses (dashed lines) against time, colored by contour. They show the same qualitative behaviour as the measured values (solid lines). (B) Comparison of the distributions of the simulated circumferences, areas, and roundnesses to their measured values (×), shown for each of the five contours and for the first timepoint.

We fit linear approximations to the experimental dynamics of $f_t^y, d_t^{\rm i}, d_t^{\rm o}$ (Figs. 4D and 4F). With these, $L_t^{\rm i}, L_t^{\rm m}, L_t^{\rm o}, A_t^{\rm i}, A_t^{\rm m}, A_t^{\rm o}$ are determined in both cases, and we find that the length ratios $L_t^{\rm o}/L_0^{\rm o}$ and $L_t^{\rm i}/L_0^{\rm i}$ increase and decrease, respectively, in the first case, and that the area ratios $A_t^{\rm o}/A_0^{\rm o}$, $A_t^{\rm i}/A_0^{\rm i}$ increase and decrease, respectively, in the second case. As discussed in the main text, these results are consistent with the experimental observations (Fig. 4G).

APPENDIX C. ADDITIONAL EXPERIMENTAL AND IMAGE ANALYSIS METHODS

1. Image fusion and deconvolution

The imaging dataset for each embryo contains three sets of images, one for each of the three temporal imaging stages discussed in the Materials and Methods section of the main text. The subset of this dataset used for contour generation includes the last timepoint in the first stage to identify the hindgut progenitors, the entire second stage involving rapid imaging of hindgut deformation, and the first timepoint in the last stage. Each stack contains 180–200 slices at a resolution of 2048 × 2048 pixels and a spatial resolution of 0.195 µm × 0.195 µm × 1 µm, totaling approximately 80 000 images for a given 130-timepoint dataset. These images were fused and deconvolved using the BigStitcher [110] Fiji [56] plugin. Embedded fluorescent beads were used to register the images and generate point spread functions. The output was a series of image stacks comprising approximately 40 000 images containing the first and last timepoint in two channels and middle timepoints only in the histone channel.

2. Surface visualizations

To construct surface visualizations, wildtype (Oregon R) embryos were collected and fixed using heat fixations as detailed in Ref. [7]. Embryos were stained with antibodies against Brachyenteron and Discs-large as a cell surface marker. Embryos were imaged by light-sheet microscopy using the live imaging and image processing protocols described above and in the Materials and Methods section of the main text. Surfaces were constructed using Imaris (Oxford Instruments, Inc.).

3. Nuclear detection and tracking

In preparation for nuclear detection, images were downsampled by 2-4× in each dimension and pixel-classified using ilastik [52]. The output of the pixel classifier is an image with identical resolution in which each pixel value corresponds to the probability of it belonging to the nuclear class. The pixel-classified images were imported into Mastodon [53], a Fiji [56] plugin built on the popular TrackMate [54] framework. Nu-

clei were detected using a difference-of-gaussians detector. The initial two-channel timepoint containing information on the Brachyenteron reporter was used to identify 350–500 nuclei within the hindgut primordium. These nuclei were tracked semi-automatedly across approximately 130 timepoints using Mastodon with manual corrections and interventions. Each nuclear track was verified manually, culminating in approximately 50 000 annotations for one embryo. Particular attention was given to nuclei in internalizing regions of the tissue, where light scatter from the yolk resulted in diminished image quality and impaired automated detection and tracking.

4. Generation of simulated errors

The semi-automated tracking means that the actual positions of the nuclear centroids could differ from the tracked points by up to a nuclear diameter. As a result, we determined the expected contribution of detection error by 1000 samplings from a uniform distribution of points within a nuclear diameter from the observed location for each nucleus at each timepoint. At each iteration, a simulated contour was generated using the approach described in the Materials and Methods section of the main text, resulting in 1000 simulated contours for each of the 5 contours at each of the 135 timepoints. Lengths, areas, and roundness of these simulated contours were calculated. The means of each of these simulated metrics for the representative embryo analyyzed in Fig. 4 of the main text are shown in Fig. S4A and reproduce the measured values qualitatively. The distribution of the simulated metrics is compared, for one timepoint, to the measured values in Fig. S4B. The errors for each metric were taken to be the standard deviation of the respective simulated metrics for each contour at each timepoint.

5. Quantification of distances between contours

The distance between contours at each timepoint was taken to be the mean of the distances between each point on one contour and the closest point on the next contour. The mean distances between the middle contour and the outermost and innermost contours are plotted against time in Fig. 4D. At each timepoint, the minor axis length f_t^x of each contour was defined to be the Euclidean distance between the two points located halfway between the initially dorsalmost and ventralmost points of the contour, as measured by their projection onto the anteroposterior axis (Fig. 4E). The major axis length f_t^y was taken to be the Euclidean distance between the initially dorsalmost and ventralmost points (Fig. 4E). These lengths were computed for each contour, although only the innermost, middle, and outermost (yellow, red, and purple) contours were used for the “coupled-ring” model.

APPENDIX D. SUPPLEMENTAL MATERIALS

1. Supplemental Figure

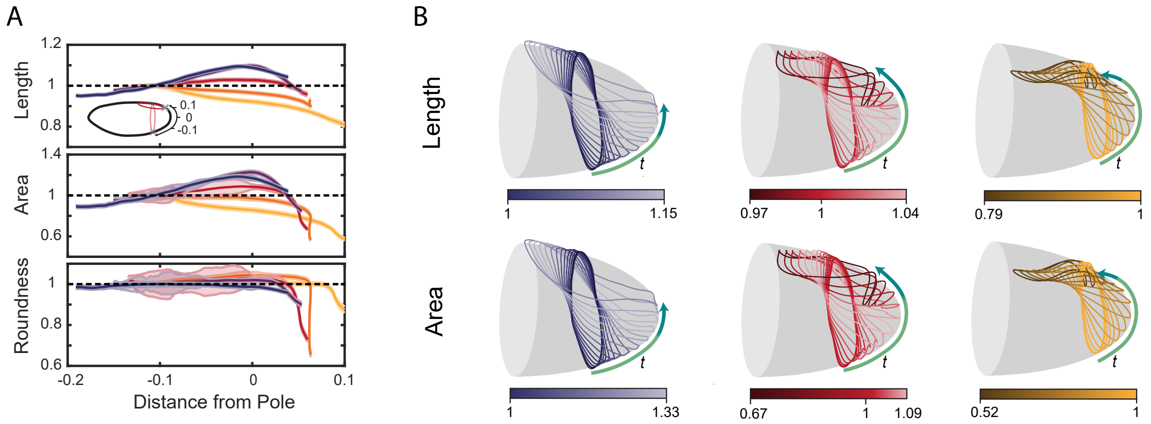

FIG. S5. Contour dynamics based on hindgut position. (A) Metrics from Fig. 4A plotted against the distance, along the surface of the embryo, of the ventralmost point of each contour; the position is normalized by arclength (inset). (B) Shapes of the outermost, middle, and innermost contours (blue, red, and yellow contours) plotted at 90 second intervals on top of a gray ellipsoid representing the surface of the embryo. The color shade of each contour indicates the changes of its length (top) or area (bottom). This shows the transient increase of the length and area of the middle and outermost contours during stage 1 as they are rotated and translated along the surface. The arrows show the direction of the movement of the ventralmost point of the contours, with the green and turquoise regions showing the positions of these points during stage 1 and stage 2, respectively.

2. Supplemental Movie

Movie S1. Contour dynamics. Contours are shown updating in time with smoothed nuclear positions visible as points. The nuclei are colored by the contour to which they belong. The movie shows the first 20 minutes of gastrulation.
